# Engineering Anti-Tumor Immunity: An Immunological Framework for mRNA Cancer Vaccines

**DOI:** 10.3390/vaccines13121222

**Published:** 2025-12-03

**Authors:** Olivia Roy, Karen S. Anderson

**Affiliations:** 1 School of Molecular Sciences, Arizona State University, Tempe, AZ 85287, USA; 2 Center for Personalized Diagnostics, Biodesign Institute, Arizona State University, Tempe, AZ 85287, USA; 3 School of Life Sciences, Arizona State University, Tempe, AZ 85287, USA

**Keywords:** mRNA vaccines, cancer immunotherapy, antigen presentation, epitope engineering, lipid nanoparticles, multi-epitope vaccine design, tumor microenvironment, precision oncology

## Abstract

The landscape of cancer immunotherapy has been redefined by mRNA vaccines as rapid clinically viable strategies that help induce potent, tumor-specific immune responses. This review highlights the current advances in mRNA engineering and antigen design to establish an integrated immunological framework for cancer vaccine development. Achieving durable clinical benefit requires more than antigen expression. Effective vaccines need precise epitope selection, optimized delivery systems, and rigorous immune monitoring. The field is shifting from merely inducing immune responses to focusing more on the biochemistry and molecular design principles that combine magnitude, polyfunctionality, and longevity to overcome tumor-induced immune suppression. We examine an integrated immunological framework for mRNA cancer vaccine development, examining how rational molecular engineering of vaccine components, from nucleoside modifications and codon optimization to untranslated regions and linker sequences, shapes immunogenicity and therapeutic efficacy. Future directions will depend on balancing combinatorial strategies combining vaccination with immune checkpoint inhibitors and adoptive cell therapies.

## 1. Introduction

The COVID-19 pandemic served as a global catalyst, transforming messenger RNA (mRNA) technology from a promising treatment strategy to a clinically validated therapeutic platform with unprecedented speed. This success has strengthened the field of cancer immunotherapy, where mRNA vaccines had been steadily advancing for over two decades [1]. By 2025, multiple clinical trials were evaluating mRNA vaccines across diverse malignancies, including melanoma, lung cancer, and intractable tumors like pancreatic ductal adenocarcinoma and glioblastoma. The clinical successes, most notably the combination of personalized mRNA vaccines with immune checkpoint inhibitors in melanoma and pancreatic cancer, have provided early-stage proof-of-concepts [2,3]. These trials have reinvigorated a core principle, that patient-specific neoantigens can elicit targeted, de novo T cell responses capable of recognizing and eliminating cancerous cells.

These pioneering successes have also highlighted some of the profound challenges that remain. Tumors are not passive targets; they are evolving ecosystems adept at creating a favorable immunosuppressive microenvironment often characterized by the loss of human leukocyte antigen (HLA) expression, increase in regulatory T cells (Tregs) and myeloid-derived suppressor cells (MDSCs), and metabolic and physical barriers that exhaust effector T cells [4,5]. As a result of this inherent heterogeneity, even a multi-antigen vaccine can face a threat of immune escape through the overgrowth of neoantigen-negative clones [6].

**Figure 1:**
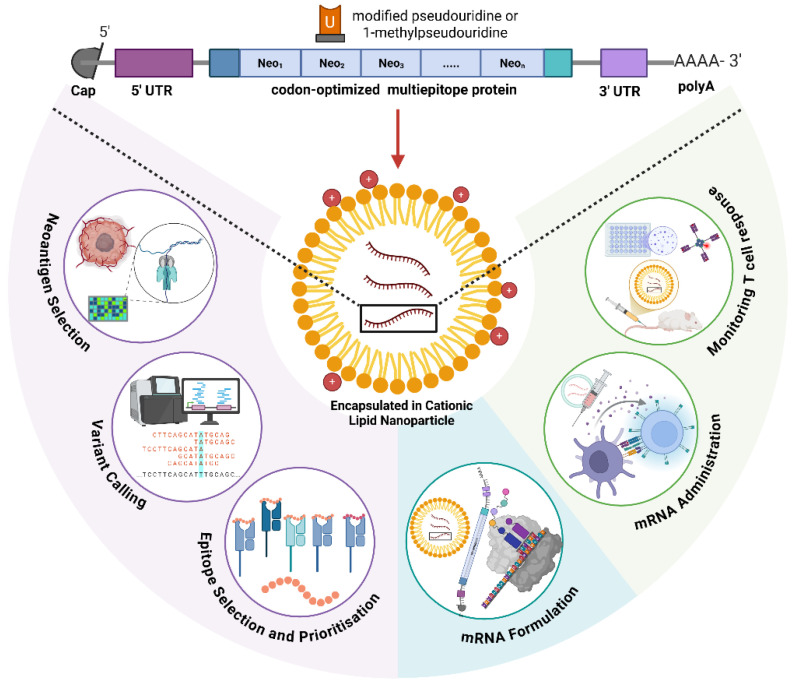
Overview of the mRNA cancer vaccine immunoengineering pipeline. A modular framework links molecular design, delivery, and immune activation to therapeutic outcome. Engineered mRNA constructs undergo codon optimization, nucleoside modification (for example, N^1^-methylpseudouridine, m^1^Ψ), and untranslated-region tuning to balance translation efficiency and innate sensing. Delivery systems—primarily lipid nanoparticles (LNPs)—enable cytosolic release in antigen-presenting cells, while emerging modalities such as polymeric nanoparticles, exosomes, and programable nucleic-acid self-assembly (DNA/RNA origami) expand precision delivery options. Following uptake, mRNA translation and antigen processing lead to MHC class I and II presentation on dendritic cells, activating CD8^+^ and CD4^+^ T cell responses. Integration with checkpoint blockade and real-time immune monitoring establishes a feedback-driven cycle for rational next-generation mRNA oncotherapy design. Abbreviations: LNP, lipid nanoparticle; HLA, human leukocyte antigen; m^1^Ψ, N^1^-methylpseudouridine; DC, dendritic cell; PLGA, poly(lactic-co-glycolicacid); APC, antigen-presenting cell; MHC, major histocompatibility complex.

The central challenge for the next generation of mRNA cancer vaccines is simply not if we can generate a T cell response but, rather, how we can precisely engineer a durable and antigen-specific response to overcome these barriers. This requires a biochemical and mechanistic approach that sees vaccine not as a single entity but as a system where each piece is engineered for the desired immune response. The mRNA is not just a messenger; its biochemical composition is a key regulator for fine tuning protein expression with innate immune activation. The chemical composition of the delivery vehicle makes it a potent adjuvant that dictates which cells are targeted and how they are activated. Figure 1 provides an overview of this systems-level perspective, outlining how engineered mRNA structure, delivery vehicle chemistry, and antigen presentation dynamics converge to dictate the quality of the antitumor immune respons. This review moves beyond a survey of the clinical landscape to propose an integrated immunological framework for the design and evaluation of mRNA cancer vaccines.

## 2. Molecular Engineering of mRNA Vaccines

### 2.1. Nucleoside Modifications: Balancing Expression and Immunogenicity

The incorporation of modified nucleosides is particularly nuanced in cancer vaccines. The immune system must be sufficiently activated to overcome tumor-associated tolerance mechanisms without triggering excessive inflammatory responses that could inhibit vaccine function or cause unacceptable toxicity. Incorporating modified nucleotides into synthetic mRNA represents a critical design decision that requires balancing antigen expression with appropriate immune activation. Endogenous mammalian mRNA contains several different types of modified nucleosides that collectively function to reduce recognition by pattern recognition receptors (PRR), an adaptation that synthetic mRNA vaccines exploit through replacement of unmodified uridine with pseudouridine derivatives [7,8]. The most extensively validated modification, N1-methylpseudouridine (m^1^Ψ) [9], used in two COVID-19 vaccines (Pfizer-BioNTech BNT162b2 and Moderna mRNA-1273) [10] reduces activation of multiple innate immune sensors, including the TLR7/8, RIG-I, and PKR-mediated translational shutdown, that would otherwise lead to rapid mRNA degradation and translational arrest [11,12]. Pseudouridine can also introduce unintended effects, including stop-codon read-through [13], generating aberrant protein variants, frameshifting^13^, and ribosomal stalling, that impede elongation rates [14]. These issues can be reduced through optimized synthesis and formulation conditions and generally do not impact vaccine performance [15]. Cancer vaccines benefit from controlled innate immune activation. Moderate stimulation promotes dendritic cell maturation, enhances antigen presentation, and provides natural adjuvant effects that are valuable in the immunosuppressive tumor environment [16,17]. Some approaches use unmodified uridine-containing mRNA to leverage the intrinsic immunostimulatory properties of single-stranded mRNA [18]. However, this strategy requires careful sequence optimization to avoid excessive type I interferon responses, which could paradoxically inhibit antigen expression through PKR activation and related antiviral mechanisms. 

Viewing nucleoside modifications as an “immunologic rheostat” highlights opportunities for fine-tuning expression efficiency and adjuvant activity across different cancers. Emerging ideas test partial nucleoside modifications, positional modifications, or even temporally controlled modification strategies to shape immune activation more precisely. Complete U→m^1^Ψ substitution generally yields strong expression with manageable immune activation, making it suitable for most applications [19,20]. These modifications do not eliminate innate immune signaling entirely. In vitro synthesized modified mRNA still triggers measurable cytokine responses upon cellular delivery, though whether this contributes beneficially to vaccine immunogenicity remains context dependent.

### 2.2. Untranslated Region Engineering: Precision Control of mRNA Function

Untranslated regions (UTRs) regulate mRNA stability, localization, and translational efficiency, making their optimization indispensable for mRNA vaccines [21]. The engineering of both 5′ and 3′ UTRs has moved beyond borrowing endogenous sequences to using machine-learning models that generate novel regulatory UTRs with enhanced stability and translation [22].

#### 2.2.1. 5′ UTR Design and Optimization

Conventionally, sequences from housekeeping genes such as α- and β-globin were selected for their short length, minimal secondary structure, and strong Kozak consensus motifs (e.g., GCC(A/G)CC AUGG) to drive translation [23,24]. These became standard in early mRNA therapeutics. Advanced capping strategies (e.g., Clean Cap) generate Cap 1 structures that improve ribosome engagement and reduce innate immune sensing triggered by cap-less or Anti Reverse Capped Analog (ARCA) capped mRNA [25,26]. More recently, use of machine-learning methods has revolutionized 5′ UTR design. For example, Chu et al. developed UTR-LM, a transformer-based language model trained on thousands of endogenous UTRs and their expression data, achieving ~30% higher protein output compared with conventional UTRs [27]. Similar models such as UTRGAN [28] and UTR-Insight [29] deliver substantial improvements in predicted ribosome loading and translation efficiency. Smart5UTR created by Tang et al. is a generative model tailored for use with m^1^Ψ-modified mRNAs highlighting that optimal UTR sequences may differ significantly depending on the specific nucleoside chemistry employed [30]. These findings emphasize that translational ability of 5′ UTR vary by nucleoside chemistry and mRNA content (e.g., m^1^Ψ-modified mRNA) by controlling ribosomal scanning, initiation site selection, and the occurrence of inhibitory secondary structures. Effective design should promote ribosome recruitment while minimizing stable hairpins that obstruct scanning, thereby reducing translation.

#### 2.2.2. 3′ UTR Engineering

The 3′ UTR critically determines mRNA half-life and translational yield by containing both stabilizing and destabilizing regulatory motifs. Optimization often removes destabilizing elements, such as AU-rich elements (AREs), which recruit degradation machinery and incorporate motifs that enhance longevity and translation [31]. High-throughput library screening of 3′ UTR variants have identified synthetic UTRs that significantly improved mRNA stability and output relative to conventional designs [32]. These expression-augmenting sequences enable synthesis of more favorable mRNA constructs tailored for vaccine applications rather than being constrained by evolutionary UTRs.

### 2.3. Poly(A) Tail Length and Tissue-Specific Control

Optimizing the poly(A) tail remains a key parameter in mRNA vaccine design. Tail lengths around 100–120 nt maximize translational efficiency by promoting mRNA circularization and ribosome recycling [33,34,35]. Engineered 3′ UTRs embed tissue-specific regulatory elements, such as microRNA-122 target sites that restrict expression in off-target tissues and enhance APC-specific translation in cancer vaccines [36]. This provides precision control of in vivo antigen expression, improving safety and immunogenicity. In Table 1, we highlight some of the strategies critical for mRNA design.

### 2.4. Codon Optimization: Multi-Parameter Molecular Engineering

Codon optimization in mRNA vaccine design represents a multi-objective challenge that extends beyond simple frequency matching to abundant tRNA species. Codon usage influences ribosomal elongation rates; abundant-tRNA codons accelerate translation while rare codons impede speed of translation. These pauses can be essential for proper co-translational protein folding; overly rapid translation may lead to misfolding or aggregation of the antigenic protein [40]. For example, systematic replacement of rare codons with optimal alternatives increased translation speed and protein yield [41] but may compromise antigen functionality without attention to protein folding. Tissue- or cell-specific codon usage patterns (e.g., muscle or immune cell expression systems) are also deployed to optimize expression-folding balance [21].

### 2.5. RNA Secondary Structure and GC Content Management

The GC content and distribution within the coding sequence significantly affect both mRNA stability and secondary-structure formation. Elevated GC (~60–70%) generally enhances stability, but GC-rich tracts especially near the 5′ region may form stable hairpins that block ribosome binding and scanning [42]. Advanced codon-optimization tools now integrate RNA-folding predictions (e.g., minimum free energy, MFE) into design, enabling reduction in inhibitory 5′-secondary structures and boosting translational output [39,43].

### 2.6. Innate Immune Recognition Motifs

Specific dinucleotide motifs can trigger unwanted innate immune responses or target mRNAs for degradation [44]. Unmethylated CpG dinucleotides, while primarily associated with DNA-based TLR9 activation, can also influence RNA stability through their recognition by zinc-finger antiviral protein (ZAP) and preferentially target CpG-rich transcripts for degradation [45,46]. In COVID-19 vaccines the SARS-CoV-2 spike protein sequence was specifically modified to reduce CpG content, as ZAP-mediated degradation could significantly reduce vaccine efficacy [47]. Similarly, UpA dinucleotides are notably underrepresented in mammalian mRNAs compared to what would be expected from random distribution. Some evidence suggests that UpA-rich sequences may activate RNase L or other innate sensors that could compromise vaccine performance [48,49,50]. Modern codon optimization algorithms therefore systematically avoid excessive CpG and UpA content while maintaining optimal codon usage and preserving critical epitope sequences.

In the context of cancer vaccines, antigen-coding sequences must preserve precise epitope peptides for immune recognition. This imposes certain immunological constraints: optimization must avoid creating cryptic splice sites, novel junctional epitopes, or disrupting post-translational modification motifs. It is imperative to actively screen for these risks while optimizing mRNA coding sequence. Modern optimization algorithms must simultaneously balance numerous competing factors, including translation efficiency, protein folding kinetics, mRNA secondary structure formation, innate immune recognition, and the preservation of critical epitope sequences essential for vaccine function.

## 3. Antigen Selection and Epitope Engineering

### 3.1. Neoantigen Discovery: Computational Pipelines and Predictive Algorithms

The accurate in silico identification of tumor-derived neoantigens is foundational to the design of mRNA-based immunotherapies. These epitopes should not only be generated by altered synonymous sequences but must effectively be processed and presented by HLA molecules and finally be recognized by T cells in sufficient numbers and affinity to drive a durable response. Most early pipelines focused primarily on MHC binding affinity; however, in reality, only a minority of high-affinity candidates triggered meaningful T cell responses, prompting the development of more novel prediction frameworks and benchmarking efforts.

#### 3.1.1. Pipeline Overview and Candidate Generation

Standard neoantigen discovery begins with whole-exome (WES) or whole-genome (WGS) sequencing, matched tumor/normal variant calling, HLA-typing, and RNA expression data. Certain pipelines add immunopeptidomic validation to confirm peptide presentation. Candidate peptides are generated from somatic non-synonymous single-nucleotide variants (SNVs), small insertions/deletions (indels), or gene fusions, typically 8–11 amino acids for MHC I and longer for MHC II. These peptides are then ranked using multiple filters that assess peptide–HLA binding, stability, antigen-processing likelihood (proteasomal cleavage, TAP transport, and HLA loading), and immunogenic potential (TCR contact residue features, self-similarity, and hydrophobicity). 

Most recent pipelines integrate features beyond binding affinity, such as allele-specific HLA internalization, mutant allele fraction/clonality, transcript abundance, peptide stability, and divergence from self. Our team helped develop one such model, HLA Inception, using convolutional networks to map the electrostatic and topological landscape of peptide–MHC binding across thousands of alleles [51]. Overall, the field is moving from binding-score ranking to mechanistically interpretable immunogenicity models like DeepHLApan that considers both immunogenicity and binding affinity [52]. Nonetheless, direct, standardized benchmarking across all these platforms remains limited. A comprehensive assessment of T cell epitope prediction is illustrated in this review article [53].

#### 3.1.2. Comparative Benchmarking of Prediction Algorithms

Despite advances, the positive predictive value (PPV) for true immunogenicity remains low. A 2023 study by Nibeyro et al. created a curated database (ITSNdb) of tumor-specific neoantigens, with validated MHC presentation and T cell response. They evaluated multiple predictors and found high-performance variability among them [54]. Another benchmark commentary underscored that most models are limited by dataset bias and sparse true positive immunogenic data [55].

Key benchmarking criteria now include:

- Binding accuracy: sensitivity/specificity of peptide–HLA binding prediction (often compared to MS–ligand or binding assay data).
- Presentation accuracy: ability to predict that a peptide will be processed and presented (proteasome/TAP/HLA loading).
- Immunogenicity recall/precision: fraction of predicted peptides that elicit T cell responses (tetramer/ELISPOT) in validation cohorts.
- Ranking power: ability to place true immunogenic peptides high in the prioritized list (e.g., top 10 % or top 20). For example, Schäfer et al. (2023) in *Bioinformatics* described ScanNeo2, a workflow integrating fusion, splicing, and SNV/indel events, and showed improved ranking performance [56].
- Allele coverage and population performance: performance across rare HLA alleles and diverse ethnicities.
- Source diversity: capacity to detect neoantigens from SNVs, indels, fusions, structural variants (SVs), and viral epitopes, e.g., Shi et al. (2023) developed NeoSV to incorporate structural variation-derived neoantigens from >2500 whole genomes [57].

Each of these algorithms have trade-offs. Binding-only predictors (e.g., NetMHCpan and MHCflurry) offer high binding score sensitivity but low immunogenicity specificity. Deep-learning models that use hydrophobicity or electrostatic embeddings (ANN Hydro and HLA Inception) improve specificity but may sacrifice sensitivity or fail on novel variant types. More complex multi-feature pipelines can better prioritize clinically relevant epitopes yet are often bespoke and lack broad benchmarking. Yao et al., 2023 described how ensemble voting classifiers improved recall but also increased false positives [58]. For viral epitopes, while binding prediction tools are well-established, immunogenicity prediction and epitope escape modeling still have limited benchmarking, especially with respect to HLA diversity and viral variant evolution.

#### 3.1.3. Long-Read Sequencing vs. Short-Read in Neoantigen Discovery

Most neoantigen pipelines rely on short-read Illumina WES/WGS and RNA-seq due to cost and assay maturity. However, these approaches lack the ability to reliably detect structural variants (SVs), gene fusions, splice isoforms, and complex rearrangements, limiting neoantigen discovery scope. Long-read technologies (e.g., Oxford Nanopore and PacBio) now offer improved detection of such complex events. Long-read sequencing can achieve haplotype-resolved variation and methylation detection [59,60]. In terms of neoantigen discovery, long reads allow full-length transcript isoform mapping, improved fusion detection, better structural variant resolution (important per the NeoSV work [57]), and allelic phasing of HLA–peptide pairs. The downside remains higher error rates (particularly older nanopore chemistry), lower throughput relative to short reads (in some cases), and higher cost per sample. As pipelines mature (e.g., “CLAE” for long-read viral applications [61]), the promise of full-length isoforms in neoantigen pipelines grows. 

Despite these advances even “gold-standard” predictors sometimes fail to out-perform generic biomarkers of tumor mutation burden (TMB) or immune infiltration [54]. Next-generation prediction platforms use machine-learning approaches that can learn from larger and more diverse datasets. Bulik-Sullivan et al. developed the Epitope Discovery using Genomics and Eluted ligands (EDGE) platform, which applies deep neural networks to datasets containing HLA-bound peptides directly eluted from 74 human tumor samples [62]. This approach achieved up to ninefold improvements in positive predictive value compared to conventional binding-based algorithms by learning from actual antigen presentation data rather than relying solely on vitro binding measurements. Future frameworks should integrate diverse variant types, including SVs/fusions (NeoSV), long-read sequencing for better variant discovery, robust immunopeptidomics validation, and broader HLA coverage. Algorithms will also need to combine binding, processing, TCR recognition, and phenotype data. 

### 3.2. Addressing Epitope Immunogenicity, Immunodominance, and Intra-Allelic Competition 

The design of a multi-epitope mRNA cancer vaccine must also address several immunological and biophysical complexities. These include maximizing epitope immunogenicity; managing immunodominance and intra-allelic competition among epitopes; selecting optimal number of epitopes and their arrangement (order, spacing, and linkers) for efficient antigen processing; and applying lessons from viral-epitope designs to tumor neoantigens.

#### 3.2.1. Predicting and Enhancing Epitope Immunogenicity

True immunogenicity of a peptide depends on multiple downstream events: peptide generation (via proteasome or other proteases), TAP transport, HLA loading and stable pMHC formation, cell-surface half-life of the pMHC, precursor T-cell frequency and TCR affinity, and the tumor or infection microenvironment [63]. In the viral-epitope realm, antigen expression kinetics and epitope abundance vary over orders of magnitude. Croft et al. (2013) [64] demonstrated in vaccinia infection that abundant pMHC does not guarantee immunodominance, with presentation onset and kinetics being critical. In mRNA cancer vaccines, this suggests the need to select epitopes not only for binding affinity but also for likely processing and presentation kinetics and to engineer the antigen such that epitope expression and liberation are optimized [65]. For example, protein engineering strategies emphasize flanking-sequence design, epitope stability, and TCR-contact residue hydrophobicity as drivers of response potency [66]. Processing kinetics also shapes both the magnitude and timing of T-cell priming. For mRNA cancer vaccines, this is critical; slower processing epitopes may miss the window of antigen-presenting cell (APC) activation or be outcompeted by faster ones. Recent reviews of cancer vaccine development stress that antigen expression kinetics and T-cell priming dynamics are central to vaccine efficacy [67].

#### 3.2.2. Immunodominance and Intra-Allelic Competition

When multiple epitopes are encoded in a single vaccine, the immune system frequently focuses on a few dominant epitopes, suppressing subdominant ones [68,69]. Immunodominance arises from competition at several levels: for proteasomal processing, for HLA loading within a given HLA allele, for presentation on antigen-presenting cells (APCs), and for T-cell clonotypes and cytokine niche space [70]. Within the same HLA allele, intra-allelic competition can occur; if two peptides bind the same HLA, the one with better processing or higher T-cell precursor availability will dominate and suppress the other. A multi-scale model by Ferretti & Kardar (2024) quantified how clonal competition and antigen dose shape immunodominance, introducing an “immunogenic space” concept [71]. For multi-epitope cassettes, reducing competition is essential. Strategies include distributing epitopes across different HLA alleles, using linker sequences to avoid interference, or avoiding clusters of very-high-affinity epitopes.

#### 3.2.3. Epitope Number Optimization and Immunodominance Management

Selecting the optimal number of epitopes in a multi-epitope vaccine entails a trade-off: too few, and the immune response may be narrow and susceptible to tumor antigen escape; too many, and the response may be diluted, subject to intra-epitope competition, or dominate in a skewed manner. Effective design considers epitope load in the context of antigen processing bottlenecks, expression kinetics, HLA diversity, and the immunodominance hierarchy [72,73]. BioNTech’s personalized neoantigen vaccines typically encode 10–20 epitopes per construct [2,74,75], while Moderna’s platforms have tested constructs containing up to 34 epitopes [76,77,78].

For mRNA cancer vaccines, immunodominance can be moderated by combining high-priority neoepitopes with selected sub-dominant but conserved epitopes to broaden coverage [79,80,81]. Distributing epitopes across multiple HLA alleles rather than concentrating on one allele [82,83]; using linkers or flanking sequences that reduce proteasomal competition; and adjusting mRNA dose to avoid saturating the APC machinery should be considered. For example, a study of multi-epitope dendritic-cell vaccines in melanoma found that combining multiple epitopes was required to inhibit tumor growth, with synergy rather than competition [84].

#### 3.2.4. Epitope Order Effects and Processing Optimization in mRNA Constructs

The ordering of epitopes, the nature of linkers, and the antigen trafficking/processing pathway profoundly influence how an mRNA-encoded multi-epitope antigen is processed and presented. Experimental and computational studies show that, when epitopes are encoded as tandem strings, the proteasome may cleave unpredictably, junctional epitopes may form, and upstream epitopes may be processed faster [85]. For mRNA vaccines, this implies that the epitope order should be optimized: placing epitopes that are more likely to be sub-dominant earlier may reduce their suppression by dominant ones; using proteasome-cleavable linkers such as AAY and GPGPG improves liberation (use of other linkers are detailed in Table 2); and avoiding back-to-back high-affinity epitopes for a single HLA allele can reduce intra-allelic competition [86,87]. Epitope ordering can shift immunodominance by placing helper-epitope (CD4+) sequences upstream of CTL epitopes to enhance APC activation and cross-priming [66]. In cancer settings, where tumor microenvironment may suppress antigen presentation, engineering constructs for rapid expression, efficient trafficking to the proteasome, and optimal pMHC stability are key.

### 3.3. Critical Design Gaps and the Road to Optimized Neoantigen Vaccines

**Figure 2:**
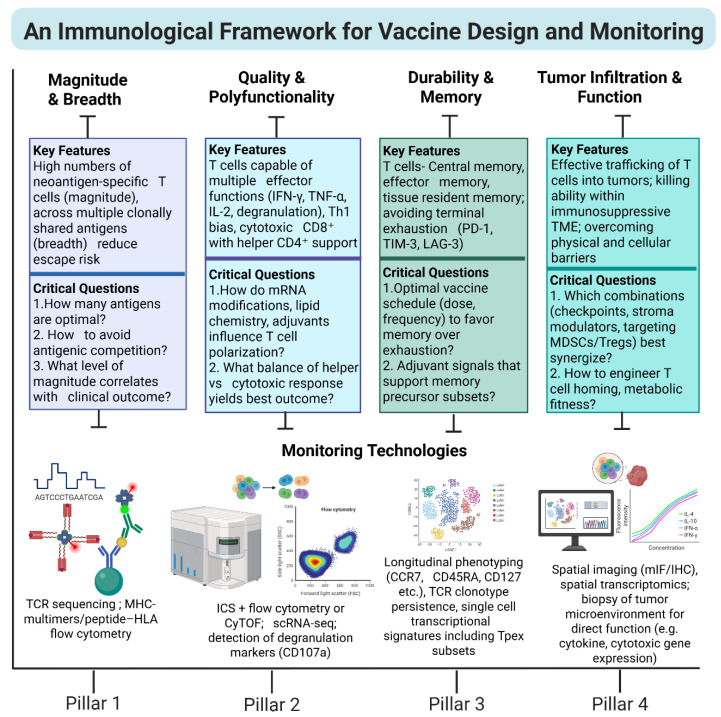
An immunological framework for mRNA cancer vaccine design and evaluation. Four measurable pillars define the potency of mRNA vaccine-induced anti-tumor immunity. This framework integrates design principles with immune monitoring to capture the multidimensional nature of therapeutic responses. (1) Magnitude and breadth—expansion of polyclonal, neoantigen-specific T cells that minimize immune escape; (2) Quality and polyfunctionality—induction of CD8^+^ cytotoxic and CD4^+^ helper T cells with coordinated cytokine secretion (IFN-γ, TNF, and IL-2); (3) Durability and memory—establishment of central, effector, and tissue-resident memory subsets sustaining long-term tumor control; and (4) Tumor infiltration and function—efficient trafficking, persistence, and cytolytic activity within the immunosuppressive tumor microenvironment. These pillars provide a unifying conceptual and analytical framework for linking molecular vaccine design parameters to functional immune readouts in clinical trials. Abbreviations: cyTOF, cytometry by time-of-flight; HLA, human leukocyte antigen; ICS, intracellular staining; IHC, immunohistochemistry; MHC, major histocompatibility complex; mIF, multiplex immunofluorescence; MDSC, myeloid-derived suppressor cells; Tregs, regulatory T cell; Tpex, progenitor exhausted T cell; TME, tumor microenvironment; TCR, T cell receptor; scRNA-seq, single-cell RNA sequencing.

Despite these advances, important limitations persist. First, predictive models for processing kinetics, linker cleavage, and TCR repertoire competition remain underdeveloped. Most immunoinformatic tools still stop at pMHC binding prediction, omitting nuanced steps of antigen processing and competition. Second, head-to-head benchmarking of multi-epitope architectures (number, order, and spacing) is limited, designing trials to compare differing architectures remains a gap. Third, tumor microenvironments may down-regulate HLA, impair APCs, or limit peptide–T-cell interactions [100,101], so even a well-designed epitope cassette may fail in vivo. Fourth, applying viral-epitope insights to the cancer neoantigen context requires caution: previous immunity, cross-reactivity, and epitope spreading are different in oncology than infection. Finally, manufacturing and scalability constraints of personalized mRNA vaccines remain a practical barrier [102]. Looking ahead, the integration of processing-kinetic models, molecular modeling, empirical immunopeptidomics of actual multi-epitope constructs, and adaptive vaccine design will be major advances. In other words, multi-epitope mRNA cancer vaccines may evolve from multiple binders to biochemically optimized immunogens, where epitope number, order, allele distribution, processing kinetics, and immunodominance are all engineered for maximal breadth, depth, and durability of T-cell immunity. As shown in Figure 2, evaluating mRNA vaccines through a structured evaluation framework enables a clearer understanding of how design choices shape response magnitude, quality, durability, and tumor infiltration.

## 4. Delivery Platform Engineering and Targeting Strategies

### 4.1. Lipid Nanoparticle Technology: Compositional Precision and Structure–Function Relationships

Lipid nanoparticles (LNPs) became the dominant mRNA delivery platform following their successful use in COVID-19 vaccines. The standard four-component architecture of standard LNPs (ionizable lipid, phospholipid, cholesterol, and PEG-lipid) has been characterized extensively in recent comprehensive reviews [103,104,105,106]. Yet these formulations exhibit fundamental limitations for cancer vaccine applications. Clinically approved LNP systems drive preferential hepatic accumulation, with ~90% of systemically administered LNPs cleared to liver within one hour [107]. However, targeted lymphoid tissue may be preferable for sustained antigen-presenting cell activation [108].

LNP internal architecture also profoundly influences mRNA delivery efficiency as observed by cryo-TEM [107] and small-angle X-ray scattering analyses [109]. Formulations enriched in bilayer lipid such as equimolar egg sphingomyelin and cholesterol demonstrate 90–100% mRNA encapsulation efficiencies while extending circulation half-life and increasing extrahepatic transfection relative to conventional formulations [107]. Formulations with LP01/cholesterol/DSPC ratios of 45:45:10 versus 73:18:9 exhibit fundamentally different lipid redistribution kinetics, promoting faster membrane destabilization and cargo release [110]. Emerging formulations, such as DOTAP-based LNPs alone or in combination, demonstrated superior oligonucleotide delivery efficiency compared to MC3 controls while exhibiting cell viability comparable to untreated cells [111,112].

### 4.2. Ionizable Lipid Chemistry: Beyond First-Generation Designs

Ionizable lipids determine LNP function through dual mechanisms; pH-dependent membrane disruption enables endosomal escape, while pattern recognition receptor engagement provides intrinsic adjuvant activity. Ionizable lipids activate NF-κB and IRF pathways via TLR4. Empty LNPs trigger activation levels nearly identical to mRNA-loaded formulations, demonstrating cargo-independent adjuvant activity [113,114]. Comparative studies of gold-standard clinical formulations SM-102 (Moderna’s mRNA-4157 and mRNA-1273) and ALC-0315 (BNT162b2) reveal transfection efficiency ranked as SM-102 > MC3 > DOTAP ≈ ALC-0315 > DODAP [115].

Biodegradable ionizable lipids incorporating ester bonds within tail regions control metabolic degradation. Ionizable and pH-responsive guanidine-based lipids (G-LNPs) facilitate efficient splenic mRNA delivery following intravenous administration with preferential antigen-presenting cell targeting. These significantly improve antigen presentation and robust T cell activation compared to conventional amine-based systems [116,117]. Sterol-conjugated ionizable lipids demonstrate luminescence levels comparable to commercial ALC-0315-LNPs while exhibiting prolonged protein expression kinetics and improved safety profiles [118]. A modular Passerini-reaction-based synthetic platform allows rapid generation of extensive biodegradable ionizable lipid libraries, with hydrogen bonding between ionizable lipids and ribose–phosphate complexes identified as critical for mRNA delivery efficiency [119]. However, critical contradictions persist; while biodegradable lipids reduce toxicity, their accelerated metabolic clearance may compromise transfection efficiency in slowly dividing immune cells. Similarly, increasing ionizable lipid content triggers RIG-I and TLR signaling pathways, resulting in elevated IL-1β and IL-6 levels necessitating precise compositional balance [113,114].

### 4.3. Overcoming Hepatotropism: Tissue-Specific Targeting Innovations

The preferential hepatic accumulation of conventional LNPs results from apolipoprotein adsorption (particularly ApoE) that directs particles toward hepatocyte LDL receptors [120]. Selective Organ Targeting (SORT) technology incorporates supplementary lipids that systematically redirect biodistribution empirically [121]. CL15H6 LNPs preferentially target splenic antigen-presenting cells [122]. Imidazole-based A3B7C2 formulations achieve 2.3-fold mRNA expression in splenic DCs and 18.3-fold enhancement when compared to SORT [123]. IR-117-17 and IR-19-Py have been benchmarked for nebulized mRNA delivery to nose and lung compared to conventional approaches [124]. BioNTech’s BNT162b2 and Moderna’s mRNA-1273 utilize phospholipid DSPC that promotes splenic accumulation instead of hepatic accumulation as seen with DOPE [103]. Charge-based targeting enables DC localization in lymphoid organs without ligand functionalization. BioNTech’s Autogene cevumeran utilizes a lipoplex structure with DOTMA and DOPE as major components. This formulation stimulates type I interferon-mediated innate immunity alongside potent adaptive responses, driving strong tumor rejection in preclinical models [125]. In pancreatic ductal adenocarcinoma, Autogene Cevumeran induced neoantigen-specific T cell responses in 8/16 patients, with vaccine responders exhibiting significantly longer recurrence-free survival versus non-responders [2]. Mannosylated LNPs delivering mRNA specifically to dendritic cells (STLNPs-Man) show not only higher uptake of mRNA in DCs but also remarkable downregulation of CTLA-4 via CD206/CD45 blockade [126]. Whether this selectivity reflects altered protein corona formation, differential receptor engagement, or modified pharmacokinetics remains unclear. Specific lipid modifications and ratios are required for reproducible lymphoid targeting different mRNA cargos and animal models have not been standardized, limiting translational predictability. These alternative compositional strategies demonstrate promising but inconsistent results when it comes to selective targeting [127].

### 4.4. PEGylation Dilemma: Anti-PEG Immunity and Alternative Stealth Strategies

PEGylated lipids provide essential colloidal stabilization but introduce immunological liabilities. Following initial LNP exposure, anti-PEG antibodies accelerate clearance of subsequent doses through complement-mediated opsonization and hepatic macrophage uptake [128]. This immune response follows a biphasic timeline with initial priming post-first dose and peak clearance effects upon subsequent administrations. Complement-activated allergy (CARPA) and severe anaphylaxis from rapid anti-PEG antibody formation have terminated multiple trials. Addition of PEG fundamentally compromises multi-dose vaccination regimens essential for durable cancer immunity [129,130,131].

Replacing PEG with zwitterionic poly(carboxybetaine) eliminates anti-PEG antibody formation while maintaining colloidal stability through super-hydrophilic properties that reduce protein adsorption and complement activation. Comparative head-to-head studies evaluating poly(carboxybetaine)-LNPs against PEG-LNPs across multiple mRNA cargos, dosing regimens, and tumor models is limited [132]. Alternative synthetic polymers like poly(2-oxazoline)s demonstrate superior mRNA translation efficiency with negligible antibody formation and have entered clinical trials for Parkinson’s disease [133,134]. Polysarcosine may also replace functionalized PEG-lipids and enable active targeting to specific cell populations [135]. Other synthetic hydrophilic polymers under investigation include poly(N-vinylpyrrolidone) (PNVP), poly(N-methyl-N-vinylacetamide) (PNMVA), and methacrylate-derived polymers (PHPMA, POEGMA, and PDMA). These alternatives show promise for biocompatibility, responsiveness, and stealth properties. Whether active targeting sufficiently compensates for anti-PEG-mediated clearance in multi-dose settings requires rigorous evaluation [136,137].

### 4.5. Beyond Lipids: Emerging Delivery Platforms and Critical Limitations

Poly(β-amino ester) (PBAE) polymers offer better biodegradability, reduced hepatotoxicity, and extended expression duration (up to four weeks versus ~1 week for LNPs) with localized expression and higher CD8 T cell responses [138,139]. Synthesis of 55 PBAE variants through monomer variation identified formulations with higher mRNA delivery efficiency than commercial LNPs and negligible liver accumulation [140]. Cell-derived exosomes (30–150 nm) offer biomimetic advantages: natural biocompatibility, intrinsic tissue targeting, and penetration of biological barriers that block synthetic carriers [141]. Current loading methods, including electroporation, transfection of producer cells, and exosome–liposome hybridization, achieve inconsistent encapsulation efficiencies, particularly for large mRNAs [142]. Microfluidic electroporation combining nano- and milli-second pulses permits production of large quantities of IFN-γ mRNA-loaded exosomes with surface-displayed CD64 adaptors for antibody-mediated targeting, achieving potent anti-glioblastoma activity against immunotherapy-resistant tumors [143].

Bacterial outer membrane vesicles have been engineered with RNA-binding protein L7Ae and lysosomal-escape protein hemolysin O for functional personalized cancer vaccines [144]. Lung spheroid-cell-derived exosomes (LSC-Exo) enable deep lung deposition and extended pulmonary retention for inhaled mRNA delivery [145]. Glioblastoma-cell-derived exosomal nanovaccines carrying endogenous tumor antigens activate APCs in lymph nodes, achieving 100% survival in GL261-luc GBM mouse models up to 5 months and preventing brain metastasis in B16F10-luc melanoma model [146]. Despite these promising results, exosome have inconsistent loading efficiency and purification challenges. In addition production scalability such as achieving clinical-grade yields (10^13^–10^15^ exosomes per patient dose) remains economically unfavorable.

mRNA-MPN nanoparticles formed by conjugation of metal ions and polyphenol complexes exhibit pH-dependent release mechanism and antioxidant and immunomodulatory effects [147]. However, significant cytotoxicity at concentrations required for efficient transfection and metal accumulation following repeated dosing raises long-term safety concerns [148]. Programable nucleic acid self-assembly can scaffold mRNA payloads, provide protective encapsulation, and facilitate cellular uptake through defined architectural features. RNA nanotechnology is used to form self-assembling RNA modules with defined nanostructures for cargo delivery. These platforms offer complementary capabilities, including tunable multivalent antigen presentation and stimulus-responsive behaviors [149,150,151,152]. However, rapid GMP manufacturing is needed, and cellular uptake mechanisms remain poorly understood. Systematic studies comparing transfection efficiency, protein expression kinetics, or immunogenicity against LNP benchmarks under identical conditions are needed.

AI-guided design of LNP formulations identified optimal compositions for pulmonary gene therapy through analysis of extensive screening datasets [153]. High-throughput platforms like SENT-seq identifies cell subtypes to effectively predict LNP uptake based on their transcriptional state in mice [154], while barcoded LNP sequencing can assess ionizable lipids for better delivery [155]. Next-generation sequencing technologies like VAX-seq support detailed profiling of vaccine-induced immune responses by analyzing genetic composition of mRNA vaccines and translation products [156].

## 5. Future Perspectives and Conclusion

### 5.1. Strategies That Work: Validated Combination Approaches

The most clinically validated approach combines mRNA cancer vaccines with immune checkpoint inhibitors such as PD-1/PD-L1 blockade. In resected high-risk melanoma, mRNA-4157 plus pembrolizumab achieved 96% overall survival at 2.5 years versus 90.2% with pembrolizumab alone. By 3 years, the combination reduced recurrence or death risk by 49% and distant metastasis risk by 62% [157,158]. This combination expands neoantigen-specific T cell populations and checkpoint inhibitors allow these T cells to overcome PD-L1-mediated suppression within tumors. The clinical evidence is emerging: cancer patients who received mRNA-based COVID vaccines within 100 days of starting immune checkpoint therapy demonstrated improved 3-year survival compared to unvaccinated patients in pilot studies [47], with survival improvements most pronounced in patients with immunologically “cold” tumors exhibiting very low PD-L1 expression [159]. Mechanistic studies reveal that mRNA vaccines stimulate PD-L1 expression within tumors, converting immunologically “cold” tumors to “hot” tumors with vigorous immune cell infiltration. Layered nanoparticle delivery systems reprogram immune systems to attack glioblastoma within 48 h, converting immunologically “cold” tumors to “hot” with vigorous infiltration [160]. 

Combining mRNA vaccines with adoptive T cell therapies amplified therapeutic responses. mRNA-based ex vivo cell engineering enables rapid CAR-T cell generation; C14-4 LNPs delivering CAR mRNA to primary human T cells achieved antitumor activity comparable to electroporation with significantly reduced cytotoxicity [161]. BioNTech’s CARVac approach (CLDN6 CAR-T + CLDN6 mRNA vaccine) in 22 patients showed enhanced CAR-T expansion and activity [162]. Novel LNPs (9322-O16B and 76-O17Se) successfully delivered CAR mRNA to macrophages and CD8+ T lymphocytes, effectively eradicating B lymphomas [163]. In vivo CAR mRNA delivery eliminates ex vivo manipulation requirements, potentially enabling rapid “off-the-shelf” cellular immunotherapy. mRNA encoding bispecific antibodies targeting CCL2/CCL5 (BisCCL2/5i) promote M1 polarization while reversing TME immunosuppression in hepatocellular carcinoma [164]. Nanovaccines combining neoantigens with macrophage-repolarizing agents downregulate M2 macrophages and Tregs while upregulating M1 macrophages and NK cells [165].

### 5.2. Next Step in mRNA Vaccine Platforms

#### 5.2.1. Circular and Self-Amplifying RNA

Beyond conventional linear mRNA, self-amplifying RNAs (saRNAs) exploit viral replicase sequences for intracellular mRNA amplification. The saRNA vaccine ARCT-154 achieves high immunogenicity at merely 5 μg dosage, 1/6th of the standard 30 μg mRNA vaccine doses, producing robust antigen expression at 5–10× lower doses than conventional mRNAs [166]. India’s approval of Gemcovac/HDT-301 in 2022, the first saRNA vaccine for use in humans, validates this next-generation platform [167]. Circular RNA vaccine studies such as circRNAOVA-luc-LNP offer stability advantages and higher translational capacity through closed-loop structures lacking 5′ and 3′ untranslated regions, protecting against exonuclease degradation and their phosphoramidate linkages [153]. Pipeline activity for circRNA and saRNA platforms increased 58.4% since January 2024, reaching 262 vaccines and therapeutics in development globally (Circular & Self-Amplifying RNA Summit, 2025). 

#### 5.2.2. Toward Integrated Precision Immunotherapy 

The FDA’s comprehensive guide “Clinical Considerations for Therapeutic Cancer Vaccines” for both early-phase and late-phase clinical trials signals readiness to meet projections of approving 10+ mRNA-based therapies by 2030. This guidance establishes standardized frameworks for trial design, endpoint selection, and regulatory submission requirements, reducing uncertainty that historically slowed vaccine development. The WHO’s mRNA Technology Transfer Programme (MTTP) expands manufacturing capabilities in low- and middle-income countries while establishing regulatory frameworks for global access. The European Medicines Agency’s alignment with FDA standards while implementing region-specific adaptations creates coordinated regulatory landscapes accelerating development timelines. Adaptive trial designs incorporating real-world data for safety and efficacy evaluation represent next-generation regulatory approaches. However, cost remains prohibitive at >USD 100,000 per patient, manufacturing timelines (reduced from 9 weeks to <4 weeks) still limit patient access before disease progression, and 50% non-responder rates indicate fundamental gaps in understanding immune activation requirements. Successful lyophilization could eliminate cold-chain logistics representing major cost and accessibility barriers, particularly in resource-limited settings [168].

### 5.3. Concluding Remarks: From Rational Design to Therapeutic Reality

With over 60 mRNA candidates in clinical development, cancer vaccines are on track to become cornerstone therapeutics. Integrated workflows are required that unite neoepitope prediction, vaccine synthesis, and quality control and current success stories reveal critical caveats. Pancreatic vaccine responder rate is about 50% (8/16 patients). We need more mechanistic understandings of non-responses with a focus on antigen selection, HLA presentation defects, TME suppression, or delivery failure. Optimal epitope number and selection remain unknown. Does more equal better, or does epitope competition limit responses? Duration of protective immunity is understudied. Most follow-up data extend to 3–4 years but can vaccine-induced responses prevent late relapses (5–10+ years)? Memory T cell persistence and recall capacity require long-term monitoring.

The goal extends beyond antigen delivery to comprehensive reprograming of anti-tumor immunity through coordinated activation of innate and adaptive pathways. This vision encompasses (1) multi-epitope constructs spanning HLA class I and II alleles mobilizing both CD8+ cytotoxic and CD4+ helper T cells; (2) incorporation of immunomodulatory factors (cytokines, co-stimulatory ligands, and checkpoint inhibitor antibodies) addressing TME immunosuppression; (3) delivery platforms ensuring mRNA cargo reaches appropriate cellular targets; (4) dosing regimens optimized through ML-guided predictive modeling; and (5) real-time immune monitoring enabling adaptive therapeutic adjustments. A successful realization of this vision could embed vaccination as a foundational component of cancer care, elevating survival, reducing recurrence, and redefining durable tumor control for patients worldwide. This positions mRNA cancer vaccines not merely as novel therapeutics but as transformative pillars of next-generation oncologic care capable of achieving sustained clinical benefit across diverse malignancies and patient populations worldwide.

## Figures and Tables

**Table 1:** Comprehensive design strategies in mRNA vaccines.

Strategy Category	Specific Approach	Mechanism of Action	Representative Examples
**5′ Cap Structure**	Cap-0 (m^7^GpppN), Cap-1 (m^7^GpppNm), Cap-2 (m^7^GpppNmNm); ARCA or co-transcriptional capping (e.g., CleanCap)	Protects mRNA from exonucleases, enhances ribosome recruitment, reduces innate immune sensing	Use of CleanCap to obtain high Cap-1/Cap-2 proportion in therapeutic mRNAs
**5′ UTR Design**	Optimized 5′ UTR sequences (e.g., human β-globin 5′ UTR)	Increases translation initiation efficiency, reduces ribosomal scanning obstacles	β-globin 5′ UTR found to enhance expression in mRNA vaccine context [37]
**3′ UTR Design**	Use of high-stability 3′ UTRs (e.g., AES + mtRNR1; human α-globin 3′ UTR)	Improves mRNA stability, lengthens translation window, decreases degradation	AES + mtRNR1 combo used in Moderna/other mRNA vaccines [32,38]
**Poly(A) Tail Length and Composition**	Optimized tail length (~100–150 nt), template-encoded or enzyme-added	Enhances transcript stability, promotes ribosome recycling, improves translation efficiency	Extended poly(A) tail designs in IVT mRNA platforms
**Coding Sequence (CDS)—Codon and Structure**	Codon optimization (CAI, tRNA abundance) + minimization of strong 5′ secondary structure	Boosts translation efficiency, reduces ribosomal pausing, improves expression and stability	Use of mRNA folding algorithms for codon/structure optimization [39]
**Combined Structural Design**	Integrated optimization of cap + 5′ UTR + CDS + 3′ UTR + poly(A)	Synergistic effect: enhanced translation, prolonged half-life, reduced unwanted innate activation.	Next-gen mRNA vaccine platforms leveraging full sequence engineering.

**Table 2:** Comprehensive linker strategies in multi-epitope vaccines.

Linker Category	Specific Sequences	Processing Mechanism	Functional Advantages	Clinical Applications	Key References
**Flexible spacers**	GGGGS, G4S variants	Non-specific spacing	Prevents steric hindrance	General epitope separation	[88,89,90]
**Proteasome-sensitive**	AAY, LKM,	Proteasomal cleavage	Enhanced MHC-I generation	Class I epitope processing	[91,92,93]
**Furin-cleavable**	RXXR, RAKR, RRRR	Furin protease recognition	Alternative processing pathway	Golgi-based processing	[94,95,96]
**2A peptides**	T2A, E2A, P2A	Ribosomal skipping	Discrete protein generation	Multi-protein constructs	[97]
**Cathepsin-sensitive**	Specific dipeptides, KK	Lysosomal processing	MHC-II pathway targeting	Class II epitope generation	[91]
**Flexible + cleavable**	GGGGS-EAAAK-GGGGS	Combined mechanisms	Optimal spacing and processing	Balanced epitope liberation	[98,99]

## Data Availability

The original contributions presented in this study are included in the article. Further inquiries can be directed to the corresponding author.

## Abbreviations

LNP	lipid nanoparticle
HLA	human leukocyte antigen
m^1^Ψ	N^1^-methylpseudouridine
DC	dendritic cell
PLGA	poly(lactic-co-glycolic acid)
APC	antigen-presenting cell
MHC	major histocompatibility complex
